# Cyberattacks Detection in IoT-Based Smart City Applications Using Machine Learning Techniques

**DOI:** 10.3390/ijerph17249347

**Published:** 2020-12-14

**Authors:** Md Mamunur Rashid, Joarder Kamruzzaman, Mohammad Mehedi Hassan, Tasadduq Imam, Steven Gordon

**Affiliations:** 1 School of Engineering and Technology, CQUniversity, Rockhampton North, QLD 4701, Australia; m.rashid@cqu.edu.au (M.M.R.); s.d.gordon@cqu.edu.au (S.G.); 2 School of Engineering, Information Technology and Physical Sciences, Federation University Australia, Gippsland Campus, Churchill, VIC 3842, Australia; joarder.kamruzzaman@federation.edu.au; 3 Information Systems Department, College of Computer and Information Sciences, King Saud University, Riyadh 11543, Saudi Arabia; 4 School of Business and Law, CQUniversity, Melbourne Campus, Melbourne, VIC 3000, Australia; t.imam@cqu.edu.au

**Keywords:** smart city, Internet of Things, cybersecurity, anomaly detection, machine learning

## Abstract

In recent years, the widespread deployment of the Internet of Things (IoT) applications has contributed to the development of smart cities. A smart city utilizes IoT-enabled technologies, communications and applications to maximize operational efficiency and enhance both the service providers’ quality of services and people’s wellbeing and quality of life. With the growth of smart city networks, however, comes the increased risk of cybersecurity threats and attacks. IoT devices within a smart city network are connected to sensors linked to large cloud servers and are exposed to malicious attacks and threats. Thus, it is important to devise approaches to prevent such attacks and protect IoT devices from failure. In this paper, we explore an attack and anomaly detection technique based on machine learning algorithms (LR, SVM, DT, RF, ANN and KNN) to defend against and mitigate IoT cybersecurity threats in a smart city. Contrary to existing works that have focused on single classifiers, we also explore ensemble methods such as bagging, boosting and stacking to enhance the performance of the detection system. Additionally, we consider an integration of feature selection, cross-validation and multi-class classification for the discussed domain, which has not been well considered in the existing literature. Experimental results with the recent attack dataset demonstrate that the proposed technique can effectively identify cyberattacks and the stacking ensemble model outperforms comparable models in terms of accuracy, precision, recall and F1-Score, implying the promise of stacking in this domain.

## 1. Introduction

Internet of things (IoT) is an interconnected scheme which promotes seamless information exchange between devices (e.g., smart home sensors, environmental sensors, automotive and road-side sensors, medical devices, industrial robots and surveillance devices) [1]. Recently, the emergence of the IoT has significantly increased its use in communities and services around the world, with the number of the linked IoT devices reaching 27 billion in 2017, and the number is projected to hit about 125 billion in 2030 [2]. IoT devices use different types of services, technologies and protocols. As a result, huge complexity will arise to maintain the future IoT infrastructures, which consequently leads to undesirable vulnerability to the system [3,4].

Since IoT devices are used in smart city applications, cyber-attacks can access in an unauthorized manner the details of citizen’s everyday activities without the knowledge of the user or administrator or reconfigure devices to an unsecured setting (e.g., in Miria botnet attack [5,6], a malware that transforms Linux networked devices remotely). In 2019, Symantec recorded a 600% rise in attacks on the IoT platform [7] where attackers tried to manipulate the linked nature of those devices.

Smart city applications pose several security challenges. Firstly, zero-day attacks can occur by exploiting vulnerabilities in different protocols in smart city applications. Secondly, is it possible to identify cyber-attacks from the network intelligently before it disrupts smart city operations? Thirdly, the IoT devices used in smart cities are resource (e.g., memory) constrained, are typically resource constrained, have limited onboard functionality for security operations and send captured data to cloud servers for processing. Existing intrusion detection systems (IDS) do not take IoT devices into account. Combining all these issues, is it is possible to design an IDS design an IDS that is tailored for IoT networks?

The data collected from the IoT system is stored on the cloud computing environment which has progressively advanced processors and adequate memory assets. However, the volume of data transmitted from the IoT terminal layer to the cloud has increased rapidly with the recent increases in IoT devices and this causes delay and congestion problems in the cloud. Fog computing is designed as a possible solution to these problems [8]. The fog layer devices can share a greater amount of computing load originally transferred to the cloud. This reduces energy consumption, network traffic and latency and removes the data storage and transmission problem. It also aims to push the computation process near the edge device, enabling a quick response to the IoT-based smart city applications. The benefits of cyber attack detection in the fog layer are two folds [9]. Firstly, the ISP or network administrator can take necessary steps to prevent large damage if attacks (e.g., infected devices) are identified early in the fog layer. Secondly, it will not interrupt the normal flow of urban life.

In the literature, some techniques (e.g., signature base techniques) have been proposed to resolve the above-mentioned issue. In the signature-based technique, a collection of previously produced signatures (attacks) are checked against the current suspicious samples [10]. If the signature extraction method is not fully able to capture the distinct feature of attacks or attack families, it may lead to misdetection of an attack or produce false alarm [11]. This technique is not suitable is not suitable for identifying unknown attacks and suffers from high processing overhead. Machine learning techniques can detect attacks during runtime and take less processing time compared to other techniques.

In this paper, we explore a machine learning-based attack and anomaly detection technique in IoT-based smart city applications. This technique is able to identify infected IoT devices which is a major challenge in the cloud computing environment [12,13]. The technique is based on the implementation of a training model in the distributed fog networks that can learn intelligently from training near to IoT layer devices and detect attack and anomaly.

A single classifier is often insufficient to develop an effective IDS, motivating researchers to build an ensemble model of classifiers. Taking a multitude of models into account, ensemble methods combine those models to generate one final model. Research has demonstrated that the ensemble model produces better performance compared to the single classifier [14]. However, there are many factors (e.g., feature selection and base classifier) that need to be considered carefully to ensure enhanced performance by the ensemble method. The most suitable ensemble techniques are bagging [15], boosting [16] and stacking [17]. In this paper, we use individual classifiers as well as ensemble techniques to achieve better IDS performance in terms of different evaluation metrics such as accuracy, precision, recall and F1-Score.

The contributions of this paper are summarized as follows:

- We explore a machine learning based attack and anomaly detection technique through analyzing network traffic in distributed fog networks over the IoT-based systems.
- Existing works have generally used signature-based techniques to detect attacks and anomalies. These techniques suffer from high overheads and are vulnerable to known threats. In this paper, we explore the feasibility of ensemble based learning as compared to single model classifiers for identifying cyberattacks in IoT-based smart city applications. Further, we consider a multi-class classification setting as compared to binary class prediction considered in most relevant works. On top of these, we consider an integration of feature selection and cross validation, which even common machine learning approaches have not been well focused in existing literature for this domain.
- Extensive evaluation incorporating the above integrations shows that the ensemble of machine learning-based classifiers works better in accurately identifying attacks and their types than single classifiers.

The paper is organized in the following sections. Section 2 discusses the related works. Section 3 discusses the IoT-based Smart city framework. Section 4 presents the proposed anomaly detection model. Section 5 presents the experimental results. Section 6 gives concluding remarks.

## 2. Related Works

In the literature, many studies have been introduced to enhance the IDS performance. In this section, we highlight the recent notable works that have used machine learning techniques as well as ensemble methods.

### 2.1. IDS Based on Machine Learning Techniques

In [18], Pahl and Aubet introduced a machine learning based technique that can predict IoT service behavior by only observing the communication between services in a distributed multi-dimensional IoT microservices in an IoT site. This technique continually learns microservice models inside in an IoT site where K-means and BIRCH based clustering techniques [19] are applied. In this case, if the cluster centers are within the three times standard deviation gap, they are grouped into the same one. The model revises cluster formation using an online learning communication model. The overall accuracy for anomaly detection by this technique is 96.5% with 0.2% false positive rate.

In [20], a joint trust light probe based defense (TLPD) mechanism was introduced to detect On and Off attack in an industrial IoT site, originated from malicious network nodes. Here, the On and Off attack meant a malicious node might target the IoT network when it is in an On or Off state. The framework was designed for the identification of anomalies using a light probe routing mechanism with the measurement of confidence estimation for each neighbor node.

Diro and Chilamkurti [10] proposed a deep learning model to detect distributed attacks in a social IoT network where they compared the performance of the deep model with a shallow neural network using the NSL-KDD [21] open source dataset that captures attack data in the distributed and centralized system. They evaluated the performance of the deep and shallow models with two-class (normal and attack) and four-class (normal, DoS, Probe, R2L and U2R) categories. For binary-class and multi-class identification, their model achieved accuracies of 99.2% and 98.27% as well as 95.22% and 96.75%, respectively, for the deep and shallow models.

In [22], Pajouh et al. proposed a two-stage dimension reduction and classification technique to detect anomaly in IoT backbone networks where they detected low frequency attacks such as user to root (U2R) and remote to local (R2L) attacks from NSL-KDD dataset because of their detrimental consequences. They used principal component analysis (PCA) and linear discriminate analysis (LDA) feature extraction method to reduce the feature of the dataset and then used naïve bayes and K-nearest Neighbor (KNN) to identify anomaly and achieved 84.82% identification rate.

In [23], Kozik et al. introduced an attack detection technique that used extreme learning machine (ELM) [24] method in the Apache Spark cloud architecture. ELM architecture and properties allow for efficient computation and analysis of the Netflow formatted data that are collected from the fog computing environment. This work concentrated on three main cases in IoT systems—scanning, command and control and infected host—and attained accuracy levels of 99%, 76% and 95%, respectively.

In [25], Hasan et al. proposed a data analysis-based method to detect attacks on IoT infrastructure which overcomes the data processing overhead of signature based techniques. Their proposed solution is able to identify and prevent the systems from attacks when it faces any irregular behavior. They performed their experiment on the publicly available IoT dataset [18]. They explored several machine learning techniques such as DT, RF, LR, SVM and ANN, among which RF classifier yielded the best results.

In [26], a random forest-based anomaly detection model was proposed that can detect infected IoT devices at distributed fog nodes. Experimenting with the UNSW-NB15 dataset [27], their binary (normal and attack) random forest (RF) classifier considered only 12 out of 49 features from the dataset. These 12 features were extracted by using ExtraTreeClassifer [28]. Performance analysis showed that they achieved 99.34% accuracy with 0.02% false positive rate.

In [29], a deep learning model was studied on NSL-KDD, UNSW-NB15, WSN-DS [30] and CICIDS 2017 [31] datasets to identify cyberattacks. They concluded that the deep learning model performs better compared to the other machine learning techniques.

### 2.2. IDS Based on Ensemble Techniques

In the literature, several ensemble methods based IDSs are proposed to enhance accuracy over base classifiers. In [32], ANN and Bayesian net based ensemble method was proposed where they used gain ratio (GR) feature selection technique and performance was evaluated on KDD’99 [33] and NSL-KDD datasets where ensemble methods achieved 99.42% and 98.07% accuracy, respectively.

In [34], Haq et al. proposed an ensemble method that combines Naive Bayes, Bayesian Net and decision tree classifier. They extracted the common features by using Best First Search, Genetic and Rank Search feature selection techniques. The ensemble technique produced 98% true positive rate when tested with 10-fold cross validation method. Gaikwad et al. [35] introduced a bagging ensemble method where they used REPTree as a base classifier. Their model achieved 81.29% accuracy on NSL-KDD dataset. In [36], Jabbar et al. proposed an ensemble method comprising alternating decision tree (ADTree) and KNN, and the performance evaluation demonstrated that the proposed ensemble achieved better detection rate (~99.8%) compared to the existing techniques.

In [37], Zhou et al. proposed feature selection and ensemble method based IDS model where a combination of correlation-based feature selection (CFS) and Bat algorithm [38] were used for optimal feature selection, followed by an ensemble method comprising DT, RF and Forest by Penalizing Attributes (Forest PA) algorithms. Experiments were performed on NSL-KDD, AWID [39] and CIC-IDS2017 datasets, achieving 99.8%, 99.5% and 99.8% accuracy, respectively.

In [40], a hybrid intrusion detection system was introduced comprising C5 classifier and One class support vector machine. The main focus of this work was to identify the common instruction and zero-day attack by using a Bot-IoT dataset [41] that contains IoT network traffic with several types of attacks. Performance analysis demonstrated that the proposed hybrid model attained higher accuracy to intrusion detection compared to Signature Intrusion Detection System (SIDS) and Anomaly-based Intrusion Detection System (AIDS).

In [42], bagging and boosting ensemble methods were proposed where the authors used decision tree and random forest tree as the base classifiers. Experiments were performed on the NSL-KDD dataset and it was found that bagging with decision trees gives better results.

Table 1 summarizes the notable works addressing intrusion and anomaly detection in networks using machine learning techniques and in some works their ensemble techniques.

Despite such a wide exploration, it is clear that different works have used different data and achieved different performance outcomes, which is not surprising due to machine learning algorithms’ often dependence on data and differing contexts may result in different outcomes. However, UNSW-NB15, the latest version of data covering intrusion detection in IOT devices, has found only relatively less exploration. In this research, we used this dataset especially considering its concurrency. Contrary to Alrashdi et al. [26], our work is not limited to binary classification or RF classifier alone. Further, in this paper, we explore the multi-class problem. In other words, our focus is not limited to only identify the normal/abnormal state of data but also to detect the exact type of attacks in fog nodes within smart city infrastructure. Our work also differs due to analyzing the performance with the base as well as ensemble classifier.

## 3. IoT-Based Smart City Framework

**Figure 1:**
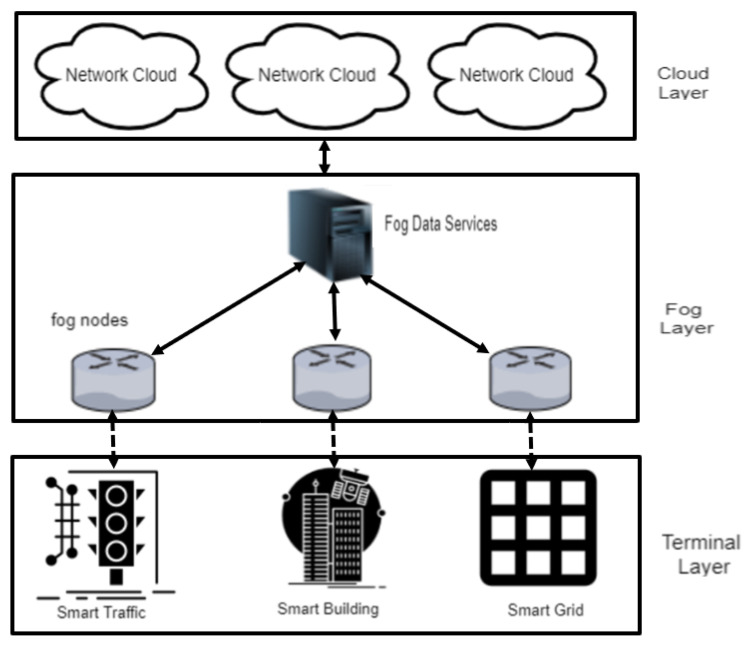
Smart city framework.

Smart city is an integrated framework where IoT technology, smart systems and information and communication technology (ICT) are collectively used to enhance the quality and performance of the different city services such as transportation, health systems, pollution control and energy distribution. A smart city framework, as based on existing literature [26], is shown in Figure 1 and consists of the following three layers: terminal layer, fog layers and cloud layer.

The cloud layer contains storage resources (e.g., servers and virtual machines) to store as well as maintain a large amount of data. The fog layer acts as a bridge between the terminal layer devices and cloud layer and is responsible to ensure the computational process and management at the edges of the network. The fog layer is more effective at identifying the different cyber-attacks than the centralized cloud layer. The terminal layer consists of a set of IoT devices (sensors) that are installed within the city to collect data.

For several reasons, IoT networks and applications are vulnerable against attacks. Firstly, most IoT devices have limited resources (e.g., small processing power and memory) and as a result suffer from limited processing capability. Secondly, IoT devices are interconnected to different protocols and the increasing number of IoT devices further causes latency in cloud centers. Thirdly, sometimes IoT devices are unattended, which makes it possible for an intruder to physically access them. Fourthly, the greater part of the data communication is wireless, exposing it to eavesdropping.

As a consequence, conventional IDS systems often fail to detect the IoT attack accurately [43]. Thus, an attacker can successfully compromise vulnerable IoT devices to connect to smart city routers and devices located at various places such as homes, shopping malls, restaurants, hotels and airports. By doing so, an attacker who compromises these IoT devices may obtain sensitive data such as information of credit card, stream video and similar personal information.

One of the key issues that smart city framework and infrastructure must ensure is its ability to deliver services in a sustainable manner to meet the needs of the current and future generations of citizens [44,45]. Some ongoing smart city projects such as those initiated in Hong Kong and Masdar city in Abu Dhabi [46] have already been criticized because of the vulnerable urban development plan and consequently doubts about sustainability of the services. The management of several facets of sustainability programs inside smart cities are facilitated by IoT, and this exposes organizations to the risks of failure from the network unavailability, security breaches and damage of IoT infrastructure from natural disasters [47]. Further, sustainable operation of services such as intelligent transportation systems, smart buildings and sustainable usage of resources such as water and energy supply, garbage disposal, etc. are highly dependent on IoT and related cyber-physical systems [48]. Machine learning techniques are used to better manage those smart city services and resources in an autonomous manner [45,49]. In addition, machine learning techniques can well detect intrusion and cyberattacks in industrial IoT [50], which therefore can enhance sustainability and ensure uninterrupted services in smart cities by thwarting attacks and intrusion on respective IoT systems. However, there is a need for more research on machine learning implementation and model verification in terms of security and privacy [51].

In this paper, we hence explore the feasibility of both ensemble-based learning and single-model classifiers for identifying cyberattacks in IoT-based smart city applications.

## 4. Proposed Anomaly Detection Model

**Figure 2:**
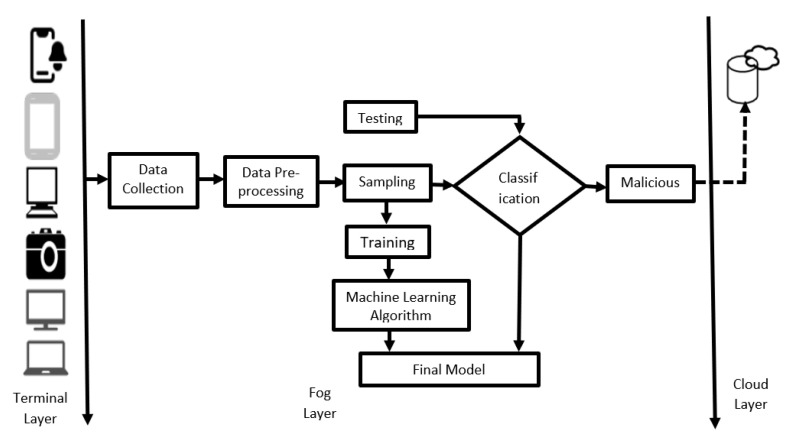
Proposal model for IoT machine learning-based IDS.

Our proposed model is shown in Figure 2. The model tracks the network traffic that goes through each fog node. Since fog nodes are closest to IoT sensors, they will be more effective at identifying the cyber-attacks at fog nodes instead of the cloud center. In this way, an attack can be quickly detected, and the IoT and network administrators can be notified of such attacks, which will then assist them to evaluate and upgrade their systems.

Notably, IDS can be categorized as host-based IDS (HIDS) and network-based IDS (NIDS). In this work, we choose anomaly-based NIDS. HIDS requires the installation of software on each network-connected device to track and identify the malicious activity focused solely on that device and is not suitable for most IoT devices which are resource constrained and support limited functionality (e.g., smart lamps, watches and lock-doors). Again, signature-based NIDS suffers from higher computational cost in storing attacks in a database and fails to detect a new attack in potential network traffic [52], which makes anomaly-based NIDS most suitable in our case. Data collected from this NIDS are used to build an ensemble of ML models to identify abnormal activities in the IoT fog networks.

### 4.1. Description of Used Datasets

We used the UNSW-NB15 [27] and CICIDS2017 [31] datasets. The reasons for using these datasets are two fold: firstly, they are relevant to the proposed smart city infrastructure concept of this paper, and, secondly, both contain samples of the recent types of attacks observed in IoT infrastructure.

#### 4.1.1. UNSW-NB15 Dataset

The UNSW-NB15 dataset [27] is a recent and highly useful IDS dataset containing the modern attacks. In 2015, the UNSW-NB15 dataset was developed to track and identify normal and attack network traffic and the raw network packets were generated by the IXIA PerfectStorm tool in the Australian Centre for Cyber Security (ACCS) cyber range lab [53]. The dataset has been preprocessed through cleaning, visualization, feature engineering and vectorization. This original dataset contains over 2.54 million samples, of which a random portion (175,341 samples) is used in our work. The considered dataset contains 56,000 and 119,241 samples, respectively, representing the benign and attack conditions. We divided our dataset into training set (140,272 samples) and test set (35,069 samples), each set containing attack and benign samples in the same ratio as the original dataset. The distribution of different attacks and anomaly across the dataset is shown in Table 2.

A brief description of the attack categories is below [27]:

- Fuzzers: It tries to cause a program or network to be suspended by feeding randomly generated data.
- Analysis: It includes various attacks of port scan, spam and html file penetrations.
- Backdoors: A security mechanism is bypassed stealthily to access a device or its data.
- Denial-of-service (DoS): The IoT network and smart city services are made inaccessible to legitimate users through malicious attempts. Distributed denial-of-service attacks (DDoS) overwhelm the target websites and online services with more traffic than the server or network can accommodate.
- Exploits: The attacker learns the security flaws in the installed software and hardware and leverages the vulnerabilities of the IoT devices and system.
- Generic: It is any technique that works against all blockciphers (with a particular block and key size), without considering the block-cipher structure.
- Reconnaissance: All actions capable of simulating assaults that gather information about vulnerabilities.
- Shellcode: It is a small code segment used as a payload to exploit vulnerability in the software.
- Worms: It replicates itself to spread to other devices and machines using connectivity.

#### 4.1.2. CICIDS2017 Dataset

The CICIDS2017 dataset was generated by the Canadian Institute for Cybersecurity (CIC) in 2017. This dataset includes benign and most recent cyberattacks, namely, DoS, DDoS, PortScan, SQL injection, Infiltration, Brute Force and Bot [31]. CICIDS2017 comprises of 2,830,743 records in eight files and each record includes 78 different features. In our experiment, we used 190,774 records where 148,777 are benign and 41,997 contain various types of attacks. Table 2 shows detailed information on sample distribution.

### 4.2. Data Pre-Processing

Feature selection is one of the key principles that greatly impacts the model’s efficacy by selecting only those features that are most relevant and thereby reduces over-fitting, improves accuracy and reduces training time. We used information gain ratio, which is a ratio of information gain to the intrinsic information proposed by Quinlan et al. [54], to select the top 25 features which are highly relevant to the prediction for both datasets. The information gain score of the features of UNSW-NB15 dataset is shown in Table 3 and Table 4. Out of 42 (UNSW-BC15) and 78 (CICIDS2017) features, the top 25 were selected based on their information gain ratio. A higher ratio for a feature can contribute more to identifying the benign and malware applications. We only consider the features whose information gain was greater than predetermined threshold 0.5 for UNSW-NB15 dataset and 0.85 for CICIDS2017 dataset.

In feature engineering phases, at first, we identify the type of features in the datasets. In UNSW-NB15 dataset, among the above mentioned 25 features, “proto” and “service” are categorical features and the rest are numerical data. This categorical data are converted into vectors. While categorical data can be translated to vectors in different ways such as ‘Label Encoding’ and ‘One Hot Encoding’, ‘Label Encoding’ [55] technique was used in this research.

### 4.3. Theoretical Consideration

Several machine learning techniques and ensemble methods were used for model building and performance evaluation. We used LR [56], SVM [57], DT [58], RF [59], KNN [60] and ANN [10] machine learning algorithms, which are widely used in the literature to design IDS scheme.

Ensemble methods are a widely used approach in machine learning that combines several base models to generate one optimal predictive model [14]. Taking a multitude of models into account, an ensemble method combines those models to generate one final model. It is based on the principle that a group of weak learners (models) comes together to form a strong learner, thereby increasing the model’s accuracy. There are three types of ensemble techniques used in the literature. Bagging [15] is a parallel ensemble technique where the base learners are generated in parallel to improve the strength and accuracy of machine learning algorithms. Boosting [16] is a sequential ensemble technique where the base learners are generated in sequence to reduce bias and variance of supervised machine learning techniques. Stacking [17] is an ensemble learning technique incorporating predictions of several base classification models into a new dataset and used as the input for another classifier which is then used to solve the problem.

### 4.4. Evaluation Criteria

In this subsection, we describe some performance matrices such as accuracy, precision, recall, F1-Score and ROC curves which are widely used in evaluating the model performance in anomaly detection applications.

These performance metrics are defined by using the following parameters:

- $t_{p}$ = true positive
- $t_{n}$ = true negative
- $f_{p}$ = false positive
- $f_{n}$ = false negative
- *p* = total positive = $t_{p}+f_{n}$
- *n* = total negative = $t_{n}+f_{p}$

Accuracy indicates the overall performance of the model with respect to both benign and attack classes and is defined as follows:

(1) $$Accuracy=\frac{t\_p+t\_n}{p+n}$$

Precision gives the information about how many selected items are relevant among the retrieved items and can be defined as follows:

(2) $$Precision=\frac{t\_p}{t\_p+f\_p}$$

Recall gives the information about how many relevant items are selected from the total number of relevant items and is defined as follows:

(3) $$Recall=\frac{t\_p}{t\_p+f\_n}$$

F1-Score can be derived from both precision and recall as follows:

(4) $$F1-Score=2\times \frac{Precision\times Recall}{Precision+Recall}$$

The Receiver operating characteristic (ROC) curve is utilized to summarize a classifier’s performance over all possible decision thresholds in a graph, and it is generated by plotting the true positive rate (tpr) against the false positive rate (fpr). Equations (5) and (6) show the calculation of true positive rate and false positive rate, respectively.

(5) $$tpr=\frac{t\_p}{t\_p+f\_n}$$

(6) $$fpr=\frac{f\_p}{t\_p+f\_n}$$

## 5. Experimental Results

Experiments were implemented using Python programming language and several libraries such as Pandas, Numpy, Matplotlib, sklearn and Keras on a HP (ELITEBOOK) laptop where the operating system was Windows 10 Education 64-bit and the processor was Intel(R) Core(TM) i5-8350U CPU @ 1.70 GHz 1.9 GHz with 16 GB RAM.

**Figure 3:**
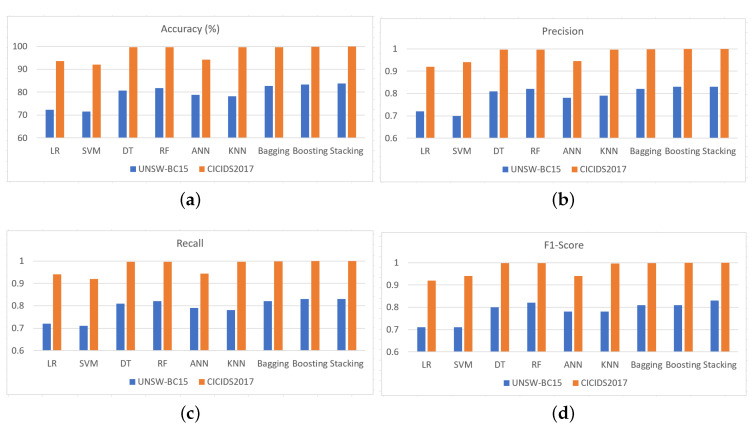
Performance evaluation of the proposed method in terms of: (**a**) accuracy; (**b**) precision; (**c**) recall; and (**d**) F1-score.

To test the performance of the base classifier as well as the ensemble classifier, 10-fold cross-validation (CV) was used where the provided dataset was randomly divided into 10 equal size subsets. Out of these 10 subsets, nine were used to build the model classifier and the remaining one was used as a test set. The same procedure was repeated ten times to ensure that each subset was used once as the test dataset. Finally, the mean accuracy summarized from each classifier in each fold was noted. Figure 3 represents different evaluation metrics for different classifiers on the training and test datasets.

We first show the performance of the different classifiers in terms of accuracy which is presented in Figure 3a. Here, the task was to classify an unknown sample into one of the ten categories for UNSW-NB15 dataset and eight categories for CICIDS2017 dataset, as shown in Table 3. For UNSW-BC15 and CICIDS2017 datasets, the accuracy of the machine learning algorithms LR, SVM, DT, RF, ANN and KNN on the test dataset are 72.32% and 93.60%, 71.49% and 92%, 80.69% and 99.7%, 81.77% and 99.7%, 78.89% and 94.2% and 78.23% and 99.7% respectively. The accuracy of ensemble methods bagging (RF as the base-learner), boosting (DT as the base-learner), stacking (base-learners of RF and ANN and Meta-learner of DT) are 82.36% and 99.7%, 83.30% and 99.8% and 83.84% and 99.9%, respectively. Among the algorithms, SVM shows poor and least performance while DT and RF shows better results compared to others. On the other hand, stacking ensemble, constructed from base- and meta-classifiers, shows better performance compared to others.

We show the performance measure in terms of precision in Figure 3b. The precision for LR, SVM, DT, RF, ANN and KNN on test dataset are 72% and 92%, 70% and 94%, 81% and 99.8%, 82%, and 99.8%, 78% and 94.5% and 79% and 99.7%, respectively. The precision of ensemble methods bagging, boosting and stacking are 82% and 99.7%, 83% and 99.8% and 83% and 99.9%, respectively. Similar to the accuracy metric, SVM shows the least precision for UNSW-NB15 dataset. However, LR shows the least precision for CICIDS2017 and RF shows better results compared to others. On the other hand, the stacking ensemble method performances better compared to others.

The performance in terms of recall is shown in Figure 3c. The recall for LR, SVM, DT, RF, ANN and KNN on test dataset are 72% and 94%, 71% and 92%, 81% and 98%, 82% and 99.8%, 79% and 94.3% and 78% and 99.7%, respectively. The recall values of the ensemble methods bagging, boosting and stacking are 82% and 99.8%, 83% and 99.9% and 83% and 99.9%, respectively. Once again, ensemble techniques yield better performance compared to the base classifier and the stacking ensemble method outperforms others. Finally, we demonstrate the performance measure in terms of F1-score in Figure 3d. The F1-score for LR, SVM, DT, RF, ANN and KNN on test dataset are 71% and 92%, 70% and 94%, 80% and 99.7%, 81% and 99.7%, 78% and 94% and 78% and 99.7%, respectively. The F1-score of ensemble methods bagging, boosting and stacking are 81% and 99.8%, 81% and 99.9% and 83% and 99.9%, respectively. Once again, ensemble techniques show better performance than base classifier and the stacking ensemble method outperforms other classifiers considered in this research.

The results show that the ensemble of learning models provides better performance than the single model classifiers on both test datasets. This implies, while existing works on the data have focused on single learning model, ensemble classifiers such as stacking represent a promising approach for application in this domain.

We also experimented with how the classifier performs when applied in a multi-class classification context. More precisely, we considered each type of attack as a separate class and then assessed the classifiers’ ability in identifying the attack from a normal situation. The results are shown in Table 5 and Table 6 for UNSW-NB15 and CICIDS2017 datasets, respectively. The results illustrate that, for various types of attack, DT and RF perform better in comparison to other algorithms. On the other hand, the stacking ensemble technique shows significant improvement compared to bagging and boosting in some cases. For example, on the UNSW-NB15 dataset, in the DoS attack, stacking yields an F1-score of 0.45 vs. 0.24 for bagging and boosting, while, in the Worm attack, these scores are 0.57, 0.37 and 0.33, respectively. In most of the other types of attacks, stacking attains an F1-score of above 0.75. On the other hand, on CICIDS2017, in the Bot attack, stacking ensemble achieved 0.950 vs. 0.898 and 0.942 for bagging and boosting.

**Figure 4:**
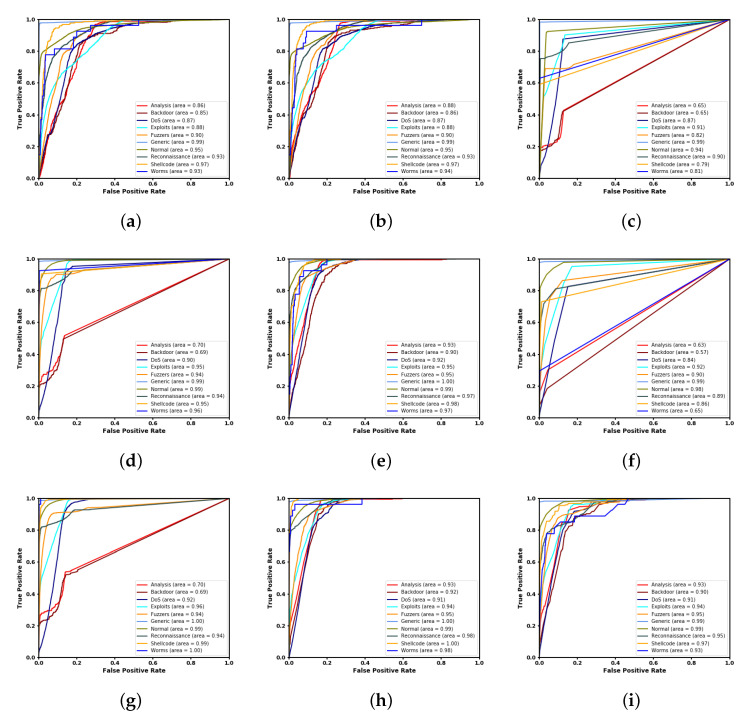
ROC Curve of: (**a**) LR; (**b**) SVM; (**c**) DT; (**d**) RF; (**e**) ANN; (**f**) KNN, (**g**) Bagging; (**h**) Boosting; and (**i**) Stacking.

Figure 4 shows the Receiver Operating Characteristic Curves for base and ensemble classifiers on UNSW-BC15 dataset. We found that, among the base classifiers, ANN shows better performance. On the other hand, among the ensemble techniques, boosting and stacking demonstrate almost the same results.

Table 7 and Table 8 show a comparison of the multi-class classification performance attained by ensemble approaches to a recent work [29] on UNSW-BC15 and CICIDS2017 datasets, respectively. The accuracies attained in our work using LR, SVM, DT, RF and KNN are 72.32% and 93.6%, 71.49% and 92%, 80.69% and 99.7%, 81.77% and 99.7% and 78.23% and 99.6%, respectively, while those in [29] are, respectively, 53.8% and 87%, 58.1% and 79.9%, 73.3% and 94%, 75.5% and 94.4% and 62.2% and 90.0%, albeit with some differences in the way the datasets were used. In [29], the researchers experimented with the boosting ensemble technique and achieved accuracies of 60.8% and 64.1 %, which are significantly lower than the accuracies attained in our work (83.3% and 99.9%) using stacking ensemble.

The results of individual classifiers as well as ensemble methods for the binary class classification are shown in Table 9 and Table 10 on UNSW-BC15 and CICIDS2017 datasets, respectively. We used the same metric as the multi-class classification. The highest accuracies of 95.45% and 99.7% and F1-scores of 95% and 99.8% by an individual classifier were achieved with RF classifier and the values of those metrics rose to 96.83% and 99.9% and 97% and 99.9%, respectively, when the stacking ensemble technique was used.

A possible reason for the proposed model’s significantly better performance compared to Vinayakumar et al. [29] is that they did not consider any feature selection. The existing work experimented with all features for both datasets. However, our proposed model considers an information gain-based feature selection technique and finally uses only 25 most important features based on their information gain ratio.

Notably, a question may be raised as to the complexity of using ensemble models as compared to a single classifier. With technological advances, however, processing units such as mobile devices are becoming increasingly faster and memory resources are becoming increasingly cheaper—a reason fog computing potentially has seen application of a wide range of algorithms including ensemble techniques [61,62]. There are also active investigation on efficient allocation of resources in fog computing [63]. Further, research has devised fog system architecture that can exploit ensemble learning without increasing latency of the system substantially [62]. Arguably, the stacking approach considered in this article can be rolled out using the architecture and efficient resource allocation mechanism. Thus, despite some increases in complexity, the finding that stacking can outperform single classifiers for counterattacks detection in IoT smart city applications has thus a notable value, especially with missing a cyberattack being linked to a high cost.

For example, the model building time for ten runs for each of the base classifiers (DT, RF and ANN) and the stacking ensemble technique on both datasets are shown in Table 11. The table shows that the model building times by the classifiers are 1.4, 2.17, 6.8 and 25.6 s for the UNSW-NB15 dataset and 5.3, 4.35, 7.4 and 27.09 s for the CICIDS2017 dataset, respectively, which shows that DT takes the least time to build the model for UNSW-NB15 while RF is the fastest for CICIDS2017. On the other hand, since the stacking ensemble model deals with more complexity by combining several base classifiers, it takes longer time to build the model for the both datasets. The time taken to test the model on a single sample by the classifiers is, respectively, 0.48, 2.53, 1.91 and 5.70 μs for UNSW-NB15 and 0.42, 1.57, 1.80 and 4.19 μs for CICIDS2017, suggesting that DT and RF take the least amount of time compared to others in both datasets. Thus, the model takes very little time, in the range of μs, to test whether an activity is malicious or not.

## 6. Conclusions

In this paper, we explore the feasibility of an ensemble based learning with single model classifiers for identifying cyberattacks within the IoT-based smart city applications. Our experiments with the most recent IoT attack database show that our ensemble approach, especially stacking, performs better than single models in identifying attacks from benign samples. Our approach employs an information gain based feature selection technique to identify the most influential features before building the model. Furthermore, in classifying attack types, our ensemble approach with stacking also leads to better performance than the single or other ensemble models used in recent works in terms of accuracy, precision, recall and F1-score metrics. Our future work will explore deep learning techniques to further enhance IoT attack detection performance.

Lastly, with automation and smart cities becoming increasingly popular, they are also increasingly being exposed to cyber threats. A denial of access or privacy intrusion within an automated system can greatly harm individual citizens and carry a substantial cost at both individual and jurisdiction levels. There can also be health risks if systems handling emergency events (e.g., accident and fire) are compromised. Our results indicating that stacking of classifiers can better detect cyberattacks in the smart city systems go beyond technical contributions and carry economic and social implications. Future research will provide further insights in this respect.

## Figures and Tables

**Table 1:** List of notable works that use machine learning (ML) and ensemble techniques for intrusion and anomaly detection. Note that not all the works deal with IoT datasets; however, they are presented here for their use of traditional or ensemble ML techniques.

Author	Year	Dataset	Type of	Method	Evaluation	IoT
			Classification		Metric	Dataset
Pahl et al. [18]	2018	Own	Multi-class	K-means	ACC = 96.3%	
Liu et al. [20]	2018	Synthetic	Multi-class	Light Probe	IR = 0.80(>)	×
				Routing		
Diro et al. [10]	2018	NSL-KDD [21]	Multi-class	Neural Network	ACC = 98.27%	×
Pajouh et al. [22]	2018	NSL-KDD	Two-tier	Naive Bayes	IR = 84.82%	×
				K-Nearest Neighbor		
Kozik et al. [23]	2018	Netflow	Multi-class	extreme learning	ACC = 99%	×
		formatted data		machine (ELM)		
Hasan et al. [25]	2019	DS2OS traffic [18]	Multi-class	LR, SVM, RF, ANN	ACC = 99.4%	
Alrashdi et al. [26]	2019	UNSW-NB15	Binary	RF	ACC = 99.34%	
Vinaykumar et al. [29]	2020	NSL-KDD,	Multi-class	Classical	ACC = 93%,	×
		UNSW-NB15,		ML Learning,	63%,	
		WSN-DS [30],		Deep	98%	×
		CIC-IDS2017 [31]		Learning	96%	
Kumar et al. [32]	2014	KDD99 [33]	Multi-class	Ensemble	ACC = 99.42%,	×
		NSL-KDD			98.07%	
Huq et al. [34]	2015	NSL-KDD	Multi-class	Ensemble	FPR = 0.0021	×
Gaikwad et al. [35]	2015	NSL-KDD	Binary	Ensemble	ACC = 81.29%	×
Jabbar et al. [36]	2017	KDD99	Multi-class	Ensemble	DR = 99.8%	×
Zhou et al. [37]	2020	NSL-KDD,	Multi-class	Ensemble	ACC = 99.8%,	×
		AWID [39],			99.5%,	×
		CIC-IDS2017 [31]			99.9%	
Khraisat et al. [40]	2019	Bot-IoT [41]	Multi-class	C5 classifier	ACC = 99.97%	
				One class SVM		
Pham et al. [42]	2018	NSL-KDD	Binary	Ensemble	ACC = 84.25%	×

**Table 2:** Distribution of normal and attack samples of the UNSW-NB15 and CICIDS2017 datasets.

Class	UNSW-NB15	Class	CICIDS2017
Normal	56,000	Benign	148,777
Analysis	2000	Bot	1964
Backdoors	1746	DoS	8000
DoS	12,264	DDoS	8000
Exploits	33,393	FTP-Patator	7938
Fuzzers	18,184	SSH-Patator	5897
Generic	40,000	PortScan	8001
Reconnaissance	10,491	Web	2180
Shell code	1133	-	-
Worms	130	-	-
Total	175,341	Total	190,774

**Table 3:** Information gain ratio for different features on UNSW-NB15 dataset.

Feature	Feature	Ratio	Feature	Feature	Ratio
Number	Name		Number	Name	
7	sbytes	1.64	27	smean	1.33
12	sload	1.268	8	dbytes	0.918
28	dmean	0.789	9	rate	0.752
35	ct_dst_sport_ltm	0.750	41	ct_sr_dst	0.733
1	dur	0.726	32	ct_state_ttl	0.707
11	dttl	0.705	3	service	0.697
36	ct_dst_src_ltm	0.695	31	ct_srv_src	0.694
2	porto	0.687	10	sttl	0.674
34	ct_src_dport_ltm	0.673	6	dpkts	0.671
13	dload	0.667	33	ct_dst_ltm	0.658
17	dinpkt	0.658	16	sinpkt	0.598
40	ct_src_ltm	0.562	25	synack	0.544
24	tcprtt	0.541			

**Table 4:** Information gain ratio for different features on CICIDS2017 dataset.

Feature	Feature	Ratio	Feature	Feature	Ratio
Number	Name		Number	Name	
53	Average Packet Size	1.1761	41	Packet Length Mean	1.16387
42	Packet Length Std	1.13817	43	Packet Length Variance	1.1381
19	Flow IAT Max	1.1175	2	Flow Duration	1.09606
37	Fwd Packets/s	1.06422	15	Flow Bytes/s	1.04735
16	Flow Packets/s	1.04504	17	Flow IAT Mean	1.03965
5	Total Length of Fwd Packets	1.01074	63	Subflow Fwd Bytes	1.01074
40	Max Packet Length	0.9991	7	Fwd Packet Length Max	0.99204
1	Destination Port	0.9871	9	Fwd Packet Length Mean	0.9729
54	Avg Fwd Segment Size	0.9729	38	Bwd Packets/s	0.95401
6	Total Length of Bwd Packets	0.90374	65	Subflow Bwd Bytes	0.90374
24	Fwd IAT Max	0.90163	21	Fwd IAT Total	0.90043
55	Avg Bwd Segment Size	0.9004	13	Bwd Packet Length Mean	0.9004
67	Init_Win_bytes_backward	0.87893			

**Table 5:** Detection of various classes in multi-class scenario on UNSW-NB15 dataset.

	Normal			Generic			Exploits			Fuzzers		
Algorithm	TPR	FPR	F1-Score	TPR	FPR	F1-Score	TPR	FPR	F1-Score	TPR	FPR	F1-Score
LR	0.81	0.04	0.85	0.98	0.02	0.97	0.70	0.12	0.64	0.70	0.008	0.55
SVM	0.81	0.052	0.84	0.98	0.013	0.97	0.72	0.121	0.64	0.66	0.09	0.53
DT	0.92	0.037	0.92	0.98	0.002	0.99	0.79	0.18	0.70	0.68	0.028	0.71
RF	0.91	0.027	0.93	0.98	0.001	0.99	0.84	0.11	0.972	0.73	0.031	0.73
ANN	0.91	0.055	0.90	0.98	0.019	0.99	0.84	0.083	0.71	0.61	0.019	0.62
KNN	0.92	0.029	0.91	0.98	0.0	0.99	0.71	0.093	0.68	0.64	0.037	0.66
Bagging	0.92	0.027	0.93	0.98	0.0	0.99	0.85	0.116	0.73	0.74	0.027	0.75
Boosting	0.93	0.035	0.93	0.98	0.001	0.99	0.83	0.112	0.72	0.69	0.029	0.71
Stacking	0.993	0.029	0.93	0.98	0.001	0.99	0.83	0.091	0.75	0.74	0.029	0.74
	**DoS**			**Reconnaissance**			**Analysis**			**Backdoor**		
Algorithm	TPR	FPR	F1-Score	TPR	FPR	F1-Score	TPR	FPR	F1-Score	TPR	FPR	F1-Score
LR	0.24	0.034	0.28	0.0	0.0	0.0	0.0	0.0	0.0	0.0	0.0	0.0
SVM	0.14	0.02	0.20	0.33	0.035	0.37	0.0	0.0	0.0	0.0	0.0	0.0
DT	0.25	0.044	0.27	0.74	0.005	0.81	0.18	0.001	0.26	0.12	0.0	0.20
RF	0.20	0.035	0.25	0.74	0.004	0.82	0.17	0.001	0.25	0.13	0.0	0.22
ANN	0.20	0.017	0.25	0.63	0.002	0.67	0.08	0.0	0.13	0.0	0.0	0.0
KNN	0.37	0.06	0.34	0.63	0.012	0.69	0.15	0.005	0.17	0.0.6	0.003	0.09
Bagging	0.19	0.03	0.24	0.74	0.005	0.82	0.14	0.0	0.23	0.11	0.0	0.19
Boosting	0.20	0.034	0.24	0.74	0.005	0.81	0.16	0.001	0.25	0.12	0.001	0.20
Stacking	0.40	0.029	0.45	0.76	0.007	0.819	0.25	0.003	0.338	0.20	0.002	0.30
	**Shellcode**			**Worm**								
Algorithm	TPR	FPR	F1-Score	TPR	FPR	F1-Score						
LR	0.0	0.0	0.0	0.0	0.0	0.0						
SVM	0.0	0.0	0.0	0.0	0.0	0.0						
DT	0.63	0.002	0.64	0.41	0.0	0.46						
RF	0.57	0.002	0.58	0.19	0.0	0.27						
ANN	0.20	0.0	0.31	0.19	0.0	0.38						
KNN	0.32	0.0	0.40	0.21	0.0	0.32						
Bagging	0.68	0.002	0.68	0.24	0.0	0.33						
Boosting	0.65	0.002	0.59	0.28	0.0	0.37						
Stacking	0.69	0.002	0.68	0.41	0.0	0.57						

**Table 6:** Detection of various classes in multi-class scenario on CICIDS2017 dataset.

	Benign			DDoS			DoS			Web		
Algorithm	TPR	FPR	F1-Score	TPR	FPR	F1-Score	TPR	FPR	F1-Score	TPR	FPR	F1-Score
LR	0.99	0.17	0.97	0.81	0.003	0.86	0.53	0.003	0.66	0.01	0.0	0.01
SVM	0.980	0.177	0.970	0.810	0.003	0.586	0.860	0.003	0.660	0.0		0.01
DT	0.995	0.003	0.995	0.995	0.0	0.995	0.996	0.0	0.995	0.997	0.0	0.997
RF	0.995	0.006	0.995	0.995	0.0	0.996	0.996	0.0	0.996	0.997	0.0	0.997
ANN	0.993	0.175	0.940	0.968	0.0	0.863	0.894	0.001	0.933	0.80	0.004	0.749
KNN	0.994	0.006	0.994	0.994	0.0	0.994	0.994	0.0	0.995	0.995	0.0	0.995
Bagging	0.999	0.006	0.999	0.999	0.0	0.999	1.0	0.0	0.999	1.0	0.0	1.0
Boosting	0.999	0.003	1.0	0.999	0.0	1.0	1.0	0.0	1.0	1.0	0.0	1.0
Stacking	0.999	0.003	1.0	0.999	0.0	1.0	1.0	0.0	1.0	1.0	0.0	1.0
Algorithm	TPR	FPR	F1-Score	TPR	FPR	F1-Score	TPR	FPR	F1-Score	TPR	FPR	F1-Score
LR	0.97	0.0	0.97	0.97	0.0	0.85	0.97	0.0	0.97	0.0	0.0	0.0
SVM	0.96	0.001	0.970	0.96	0.014	0.850	0.96	0.001	0.960	0.0	0.0	0.0
	**PortScan**			**FTP-Patator**			**SSH-Patator**			**Bot**		
DT	0.98	0.0	0.98	0.98	0.0	0.99	0.98	0.0	0.98	0.925	0.001	0.925
RF	0.98	0.0	0.98	0.98	0.0	0.99	0.98	0.0	0.98	0.884	0.001	0.897
ANN	0.600	0.0	0.746	0.80	0.002	0.870	0.80	0.001	0.878	0.628	0.0	0.768
KNN	0.97	0.0	0.97	0.97	0.0	0.97	0.98	0.0	0.98	0.883	0.002	0.871
Bagging	1.0	0.0	1.0	1.0	0.0	1.0	1.0	0.0	1.0	0.878	0.001	0.898
Boosting	1.0	0.0	1.0	1.0	0.0	1.0	1.0	0.0	1.0	0.935	0.001	0.942
Stacking	1.0	0.0	1.0	1.0	0.0	1.0	1.0	0.0	1.0	0.950	0.001	0.950

**Table 7:** Comparison of multi-class classification performance on UNSW-NB15.

	Proposed Method				Existing Method [29]			
**Algorithm**	**Accuracy**	**Precision**	**Recall**	**F1-Score**	**Accuracy**	**Precision**	**Recall**	**F1-Score**
LR	0.7232	0.72	0.72	0.71	0.538	0.414	0.538	0.397
SVM	0.7149	0.70	0.71	0.70	0.581	0.586	0.581	0.496
DT	0.8069	0.81	0.81	0.80	0.733	0.721	0.733	0.705
RF	0.8177	0.82	0.82	0.82	0.755	0.755	0.755	0.724
ANN	0.7889	0.78	0.79	0.78	-	-	-	-
KNN	0.7823	0.79	0.78	0.78	0.622	0.578	0.622	0.576
Bagging	0.8263	0.82	0.82	0.81	-	-	-	-
Boosting	0.833	0.83	0.83	0.81	0.608	0.502	0.608	0.526
Stacking	0.8384	0.83	0.83	0.83	-	-	-	-

**Table 8:** Comparison of multi-class classification performance on CICIDS2017.

	Proposed Method				Existing Method [29]			
**Algorithm**	**Accuracy**	**Precision**	**Recall**	**F1-Score**	**Accuracy**	**Precision**	**Recall**	**F1-Score**
LR	0.936	0.92	0.94	0.92	0.870	0.889	0.870	0.868
SVM	0.92	0.94	0.92	0.94	0.799	0.757	0.799	0.727
DT	0.997	0.997	0.997	0.997	0.940	0.965	0.940	0.949
RF	0.997	0.997	0.997	0.998	0.944	0.970	0.944	0.953
ANN	0.942	0.945	0.943	0.940	-	-	-	-
KNN	0.996	0.997	0.997	0.997	0.909	0.949	0.909	0.922
Bagging	0.997	0.998	0.998	0.998	-	-	-	-
Boosting	0.998	0.999	0.999	0.999	0.641	0.691	0.641	0.653
Stacking	0.999	0.999	0.999	0.999	-	-	-	-

**Table 9:** Comparison of binary classification performance on UNSW-NB15.

	Proposed Method				Existing Method [29]			
**Algorithm**	**Accuracy**	**Precision**	**Recall**	**F1-Score**	**Accuracy**	**Precision**	**Recall**	**F1-Score**
LR	0.9173	0.92	0.92	0.92	0.7430	0.955	0.653	0.775
SVM	0.9197	0.92	0.92	0.91	0.653	0.998	0.492	0.659
DT	0.9502	0.95	0.95	0.95	0.897	0.982	0.864	0.919
RF	0.9545	0.95	0.95	0.95	0.903	0.998	0.867	0.924
ANN	0.940	0.94	0.94	0.94	-	-	-	-
KNN	0.9437	0.94	0.94	0.94	0.81	0.926	0.905	0.915
Bagging	0.9574	0.96	0.96	0.96	-	-	-	-
Boosting	0.9562	0.95	0.95	0.95	0.90	0.985	0.866	0.922
Stacking	0.9683	0.97	0.97	0.97	-	-	-	-

**Table 10:** Comparison of binary classification performance on CICIDS2017.

	Proposed Method				Existing Method [29]			
**Algorithm**	**Accuracy**	**Precision**	**Recall**	**F1-Score**	**Accuracy**	**Precision**	**Recall**	**F1-Score**
LR	0.936	0.92	0.94	0.92	0.839	0.685	0.850	0.758
SVM	0.92	0.94	0.92	0.94	0.799	0.992	0.328	0.493
DT	0.997	0.997	0.997	0.997	0.935	0.839	0.965	0.898
RF	0.997	0.997	0.997	0.998	0.940	0.849	0.969	0.909
KNN	0.997	0.998	0.998	0.998	0.910	0.786	0.968	0.865
Bagging	0.998	0.999	0.999	0.999	-	-	-	-
ANN	0.940	0.94	0.94	0.94	-	-	-	-
Boosting	0.998	0.999	0.999	0.999	0.914	0.887	0.918	0.902
Stacking	0.999	0.999	0.999	0.999	-	-	-	-

**Table 11:** Mean model building time and per sample test time for the base and Stacking classifier on UNSW-BC15 and CICIDS2017 datasets. The value within the bracket indicates the standard deviation among ten trails.

	UNSW-NB15		CICIDS2017	
**Algorithm**	**Model Build Time**	**Test Time**	**Model Build Time**	**Test Time**
	**(s)**	**(** μ **s)**	**(s)**	**(** μ **s)**
DT	1.4 (±0.0017)	0.48	5.3 (±0.0044)	0.42
RF	2.17 (±0.0019)	2.53	4.35 (±0.0003)	1.57
ANN	6.8 (±0.0044)	1.91	7.4 (±0.0055)	1.80
Stacking	25.6 (±0.0017)	5.70	27.09 (±0.0040)	4.19
